# Parental feeding perceptions and family health behaviours: an analysis based on nutrition and physical activity tendencies

**DOI:** 10.3389/fped.2025.1704116

**Published:** 2026-01-16

**Authors:** Bekir Erhan Orhan, Aydın Karaçam, Walaa Jumah AlKasasbeh, Adam Tawfi Amawi, Umut Canli

**Affiliations:** 1 Faculty of Sports Sciences, Istanbul Aydın University, Istanbul, Türkiye; 2 Faculty of Sports Sciences, Bandırma Onyedi Eylül University, Balıkesir, Türkiye; 3 Department of Physical Education, School of Sport Sciences, The University of Jordan, Amman, Jordan; 4 Department of Movement Sciences and Sports Training, School of Sport Sciences, The University of Jordan, Amman, Jordan; 5 Faculty of Sports Sciences, Tekirdağ Namık Kemal University, Tekirdağ, Türkiye

**Keywords:** behaviours, family health, home environment, parental feeding practices, physical activity

## Abstract

Childhood obesity is a growing public health concern with long-term physical and psychological consequences. Parents, as primary caregivers, play a central role in shaping children's eating habits and physical activity behaviours. This cross-sectional study examined how parental feeding perceptions and practices relate to family nutrition and physical activity patterns, and whether these associations vary according to parental demographic characteristics, particularly age. The sample comprised 268 parents of children aged 8–11 years in Türkiye, recruited from primary schools using purposive sampling. Data were collected via the Child Feeding Questionnaire (CFQ) and the Family Nutrition and Physical Activity Screening Tool (FNPA), both of which showed acceptable internal consistency in this sample. Pearson correlations, one-way ANOVA, and multiple linear regression with effect size estimates were used to analyse the data. Parental age showed no significant correlations with most CFQ subscales, except for a small negative association with restriction (*r* = –0.12, *p* < .05), indicating that older parents report slightly lower use of restrictive feeding practices. General obesity-related parental perceptions (perceived parent and child weight, concern about child's weight) were not significantly related to FNPA scores, and differences in CFQ and FNPA scores by parental gender, education level, and child age were statistically non-significant and negligible in magnitude. In contrast, FNPA scores were positively associated with parental responsibility (*r* = 0.32, *p* < .01) and monitoring (*r* = 0.41, *p* < .01). Regression analysis showed that higher responsibility and monitoring predicted more favourable family nutrition and physical activity patterns, whereas restriction was a negative predictor, with the model explaining approximately one quarter of the variance in FNPA scores. These findings underscore the importance of strengthening parental responsibility and monitoring, rather than restrictive feeding, in interventions designed to promote healthier lifestyle patterns in middle childhood.

## Introduction

Childhood obesity is widely recognised as one of the most pressing public health challenges of the 21st century (1, 2). The prevalence of overweight and obesity in children has risen sharply over recent decades, affecting not only high-income countries but also rapidly transitioning economies where urbanisation, reduced daily movement, and dietary shifts have altered traditional living patterns (3–5). The consequences extend far beyond appearance; excess weight in childhood is associated with cardiometabolic and musculoskeletal problems, early pubertal development and psychological difficulties such as low self-esteem, body dissatisfaction and depressive symptoms (6, 7). Many of these complications may persist into adulthood, increasing the risk of chronic disease, premature mortality and reduced quality of life (8, 9). Childhood obesity is shaped by the interplay of biological, behavioural, environmental and socio-cultural influences (10–12). Within this constellation, the family environment plays a central role in shaping children's eating habits, physical activity routines and health-related beliefs (13, 14). Parents are key agents in providing access to nutritious foods and opportunities for movement, modelling everyday habits, setting expectations and reinforcing health-related values; their attitudes toward body weight, their evaluations of the child's health and their responses to weight-related concerns can meaningfully influence children's emerging self-regulation and lifestyle patterns (15–17).

**Table 1:** Demographic characteristics of parents according to gender, child’s gender and education level.

Variable	Category	*N*	%
Parental gender	Female	101	37.7
	Male	167	62.3
	Total	268	100.0
Child's gender	Girls	105	39.2
	Boys	163	60.8
	Total	268	100.0
Parents' education	Middle school	85	31.7
	High school	102	38.1
	Bachelor's degree	81	30.2
	Total	268	100.0

Parental perceptions of a child's weight and eating patterns are therefore a crucial component of family health dynamics and can influence children's long-term well-being (16, 18). Recognition of excess weight and the degree of concern expressed may shape the strategies parents adopt, including monitoring dietary intake, encouraging physical activity or imposing food restrictions (19, 20). Although such strategies often arise from a genuine wish to protect the child's health, they can also have unintended consequences, including disordered eating behaviours, body image disturbance, heightened anxiety around food and reluctance to engage in physical activity (19, 21). These effects depend not only on what parents do but also on how they do it—whether their approach is experienced as controlling, supportive or disengaged. Over time, the consistency, emotional tone and perceived intent of parental practices can influence the internalisation of health-related values, children's self-perceptions and their relationship with food and movement (22, 23). Understanding the nuances of parental perceptions and responses is therefore critical for designing family-based strategies that promote healthy habits without increasing psychological burden.

More broadly, parents are central in shaping children's health-related attitudes and behaviours, particularly in relation to eating habits and everyday physical activity (14, 24, 25). In day-to-day family life, they plan meals, decide which foods and drinks are available at home, organise mealtimes and snacks, and determine the extent to which screen use and active play are incorporated into children's routines (14, 24, 26, 27). At the same time, the comments they make about food, body weight and lifestyle, and the priorities they model in their own eating and activity choices, send powerful indirect messages about what is considered “normal” or desirable within the household. The patterns that children repeatedly observe and experience in this family context often provide the basis for their later nutrition and physical activity habits (28, 29), underscoring the importance of understanding parental feeding perceptions and everyday family health behaviours together rather than in isolation. When parents consistently support balanced nutrition and regular physical activity within a structured yet responsive climate, children are more likely to adopt and maintain these habits (30, 31). In contrast, inconsistent, overly restrictive or highly permissive approaches may generate confusion or resistance and undermine the development of self-regulation (14, 32). Consequently, understanding parental attitudes, perceptions and everyday practices is essential for clarifying processes that may either mitigate or amplify risk for unhealthy lifestyle patterns in early life. Public health strategies that prioritise the family environment, and particularly prevention-oriented approaches, are likely to be especially important. In this context, the present study does not aim to estimate obesity prevalence or diagnose obesity; instead, it focuses on parental feeding perceptions and family health behaviours within the home environment.

Although a growing body of research has examined parental perceptions of childhood overweight and specific feeding practices, much of this work has focused on children's anthropometric outcomes or isolated eating behaviours rather than broader family-level lifestyle configurations that integrate both nutrition and physical activity. Evidence from middle-income and rapidly urbanising settings, such as Türkiye, remains relatively limited despite marked changes in dietary patterns, sedentary behaviour and parenting roles in recent years. Existing studies frequently do not examine parental weight perceptions, concrete feeding strategies and the home environment for nutrition and physical activity within a single analytical framework. Addressing this gap is important for understanding how everyday parental practices co-occur with more health-promoting or more obesogenic family routines in specific cultural contexts.

In this context, the present study aimed to examine how parental perceptions and feeding practices related to childhood weight and eating are associated with family nutrition and physical activity patterns among parents of primary school children in Türkiye. Specifically, the study investigated (a) the relationships between parental perceptions, feeding practices and family nutrition and physical activity patterns, (b) whether these associations differ according to selected demographic characteristics and (c) the extent to which feeding practices are associated with variation in family nutrition and physical activity patterns. It was expected that responsibility and monitoring would be positively associated with more favourable family environments, whereas more controlling practices, such as restriction and pressure to eat, would show weaker or negative associations with health-promoting family routines.

## Method

### Model

This study, which aims to examine the relationships among parents' perceptions and feeding practices, families' tendencies toward nutrition and physical activity, and some demographic variables, uses a descriptive relational screening model. Although relational studies do not establish causality, they can identify associations between variables and generate hypotheses for future longitudinal or experimental research (33).

### Study group

Since the study included parents of children aged 8–11 years, a purposive sampling strategy was used to recruit parents who met these inclusion criteria. In purposive sampling, the researcher has a specific purpose or objective in mind when selecting the sample. Therefore, the sample is selected based on the characteristics or attributes that the researcher is interested in studying (60). Of the 268 parents selected through purposive sampling, 37.7% (*n* = 101) were female and 62.3% (*n* = 167) were male. 31.7% (*n* = 85) of the parents were middle school graduates, 38.1% (*n* = 102) were high school graduates, and 30.2% (*n* = 81) were university graduates. Among the children of the participating parents, 39.2% (*n* = 105) were girls and 60.8% (*n* = 163) were boys. The mean age of the participating parents was 41.1 years. Participants resided in Istanbul and neighbouring districts; therefore, the sample reflects this regional urban context rather than being nationally representative of all parents in Türkiye. Data were collected via a self-administered online questionnaire that parents completed independently. Recruitment took place through informal parent networks and publicly accessible online platforms for parents of primary school children (e.g., social media groups and messaging applications), and not through official school communication channels, teacher-mediated announcements or school mailing lists. No information was obtained from school records or directly from children. Inclusion criteria were: (a) being a parent or primary caregiver of a child aged 8–11 years who was enrolled in a primary school in Türkiye at the time of the study and (b) providing informed consent. Parents who did not complete the questionnaire in full were excluded from the analysis.

### Data collection tools

In the study, the Turkish adaptation of the Child Feeding Questionnaire (CFQ) by Erdim et al. (34) and the Family Nutrition and Physical Activity Screening Tool (FNPA) developed by Özdemir et al. (35) were used. A personal information form, including demographic information such as the age of the parents, the age of the children, and the level of education of the individuals, was created to determine these characteristics (see Table 1).

### The child feeding questionnaire (CFQ)

In this study, the Turkish adaptation of the Child Feeding Questionnaire (CFQ) by Erdim et al. (34) was used. Based on information provided by parents of children aged 2–11, the CFQ is a tool that evaluates perceptions, concerns, and practices related to childhood obesity and feeding behaviours. The questionnaire was developed to determine parents' perceptions, concerns, and practices related to child feeding, as well as the relationship between these practices, children's eating patterns, and the control of food intake in relation to obesity. The original CFQ was developed in 1994 by Johnson and Birch (36) based on Costanzo and Woody's (37) theory regarding parenting behaviours related to children's predisposition to obesity, and it initially consisted of 24 items. In 2001, Birch et al. (38) revised the CFQ, increasing the number of items to 31. All items are rated on a Likert-type scale ranging from 1 to 5. The revised CFQ consists of 31 items and 7 subscales. Four of these subscales evaluate parents' perceptions and concerns about their children's predisposition to obesity, while the other three subscales assess parents' feeding practices toward their children. The questionnaire does not provide a total score; instead, each subscale is scored independently (38).

In the Turkish adaptation study of the CFQ conducted by Erdim et al. (34) in 2017, the Cronbach's alpha coefficients for the subscales were as follows: “Perceived Responsibility (PR)” subscale = 0.59, “Perceived Parent Weight (PPW)” subscale = 0.70, “Perceived Child Weight (PCW)” subscale = 0.77, “Concern about Child's Weight (CCW)” subscale = 0.72, “Restriction (RST)” subscale = 0.79, “Pressure to Eat (PE)” subscale = 0.70, “Monitoring (MNT)” subscale = 0.81, and 0.82 for the entire scale. For the present sample, Cronbach's alpha coefficients were 0.69 for the PR subscale, 0.70 for PPW, 0.78 for PCW, 0.69 for CCW, 0.76 for RST, 0.73 for PE, 0.90 for MNT, and 0.83 for the total scale.

Significant positive correlations were found between test–retest scores for all seven subscales (PR: *r* = 0.41, *p* = 0.000; PPW: *r* = 0.64, *p* = 0.000; PCW: *r* = 0.63, *p* = 0.000; CCW: *r* = 0.64, *p* = 0.000; RST: *r* = 0.54, *p* = 0.000; PE: *r* = 0.58, *p* = 0.000; MNT: *r* = 0.57, *p* = 0.000). The Kaiser–Meyer–Olkin (KMO) test result was 0.75. The confirmatory factor analysis (CFA) indicated that the fit indices were within acceptable to good levels (*χ*^2^/*df* = 3.07, GFI = .86, CFI = .91, AGFI = .83, RMSEA = .06, SRMR = .07).

### Family nutrition and physical activity scale (FNPA)

The Family Nutrition and Physical Activity Screening Tool was developed in 2009 by Ihmels et al. (39) at Iowa State University in collaboration with the American Dietetic Association (ADA). The Turkish adaptation of the scale was carried out by Özdemir et al. (35) in 2022. The Turkish version of the scale consists of 20 items and is evaluated using a 4-point Likert scale. Each item is rated on a scale of 1 (never/almost never), 2 (sometimes), 3 (often), and 4 (very often/always). Six items (items 3, 4, 5, 7, 10, and 13) are reverse-coded. The total score ranges between 20 and 80. In addition to the total score, the scale includes ten domains reflecting specific aspects of the family environment (family meals, family eating habits, food choices, beverage choices, restriction/reward, screen time, healthy environment, family activity, child activity and family schedule/sleep routines). For the present study, domain scores were computed as the mean of the items belonging to each domain, with higher scores indicating more health-promoting family nutrition and physical activity patterns in that domain. In the present study, higher total scores were interpreted as reflecting more health-promoting family nutrition and physical activity routines overall, whereas lower scores indicated less favourable patterns in these domains. The scale was used to characterise family nutrition and physical activity patterns rather than to classify children according to obesity risk.

In analyses conducted by Özdemir et al. (35) in 2022, the Kaiser–Meyer–Olkin (KMO) statistic was 0.78, indicating that the sample was suitable for factor analysis of the FNPA items. Bartlett's test of sphericity (*χ*^2^ = 2821.2, *p* < 0.001) showed that the data were appropriate for exploratory factor analysis. The internal consistency (Cronbach's alpha) for the entire scale was 0.76, and the test–retest reliability correlation coefficient was 0.92. In the analyses conducted for the present study, Cronbach's alpha was calculated as .73 for the total scale, .77 for Physical Activity (PA) (Factor 1), .69 for Parental Behaviours (PB) (Factor 2), .60 for Unhealthy Eating Habits (UEH) (Factor 3), .70 for Healthy Food Intake (HFI) (Factor 4) and .70 for Sedentary Behaviours–Restriction/Reward (SB-RR) (Factor 5). Confirmatory factor analysis was used to test the model derived from the exploratory factor analysis. The fit indices indicated that the 20 items of the FNPA adequately represented the item responses in Turkish parents: *χ*^2^/*df* = 2.22, *χ*^2^ = 356.60, *df* = 160, *p* < 0.001, CFI = 0.95, RMSEA = 0.04, NFI = 0.92, and GFI = 0.95 (35, 40).

### Ethical approval and consent

The study was approved by the Istanbul Aydın University Social and Human Sciences Ethics Committee (Approval No: 2025/7). All participants provided electronic informed consent, and procedures complied with the Declaration of Helsinki.

### Data analysis

In the data analysis, the dataset was initially examined for incorrect values, outliers, normality, and multicollinearity. It was observed that there was no incorrectly entered data. Data analysis was conducted using SPSS 27 software. In addition to descriptive statistics, distributions were examined for normality using skewness and kurtosis tests. Since the skewness and kurtosis coefficients were between −1.5 and +1.5, the distribution was considered normal (41). For pairwise comparisons, independent samples *t*-tests were used, and for multiple comparisons, ANOVA with Tukey *post hoc* tests was applied. Pearson's product-moment correlation coefficient was used to determine relationships between variables. The effects of CFQ subscales on FNPA scores were examined using multiple linear regression analysis. The significance level was set at *p* < .05. In addition to significance testing, effect sizes were calculated and reported (Cohen's *d* for *t*-tests, eta squared (*η*^2^) for ANOVA, and Pearson's r for correlations). Effect sizes were interpreted as small, medium, or large according to Cohen's (42) guidelines. In regression analyses, it is methodologically appropriate to use only subscale scores as independent variables for instruments that lack a theoretically supported total score, while including total scores as dependent variables for instruments designed for interpretation at the total-scale level. The literature emphasises that entering both the total score and the subscales of the same instrument into the same regression model can lead to multicollinearity problems and reduce the interpretability of the findings (41, 43). For this reason, using the total score of an instrument as the dependent variable and the instrument's subscales without a total score as independent variables is a valid and widely applied analytical strategy (44).

### Findings

Table 2 shows that, for responsibility and monitoring, mean scores were above the theoretical midpoint of the possible range, indicating that many parents reported an active role in overseeing their children's eating. At the same time, scores for restriction and pressure to eat were also above the midpoint, suggesting that controlling feeding strategies were relatively common in this sample. The overall family nutrition and physical activity score reflected a generally favourable, although not optimal, home environment.

**Table 2:** Descriptive statistics for CFQ subscales and FNPA scores (*n* = 268).

Variable	Minimum	Maximum	Mean	SD
PR	3.00	15.00	11.35	2.77
PPW	5.00	18.00	11.62	2.24
PCW	5.00	25.00	14.54	2.99
CCW	3.00	15.00	8.94	2.90
RST	8.00	40.00	25.49	6.67
PE	4.00	20.00	12.44	4.16
MNT	3.00	15.00	11.02	3.35
FNPA Total	37.00	77.00	59.42	6.78
PA	5.00	20.00	15.38	3.11
PB	5.00	16.00	11.33	1.92
UEH	4.00	16.00	11.17	2.19
HFI	5.00	12.00	9.80	1.69
SB–RR	6.00	16.00	11.72	1.93

No significant differences were observed in parental feeding practices or in family nutrition and physical activity patterns according to parent gender or child gender (all *p* > .05). Effect sizes for these comparisons were in the negligible to small range, indicating that, in practical terms, mothers and fathers, and parents of girls and boys, reported broadly similar patterns.

Table 3 shows that parents' perceptions and concerns about childhood obesity differed significantly by education level only in parental responsibility subdimension of the CFQ [*F*(2,265) = 3.13, *p* = .04, *η*^2^ = 0.023]. According to the *post hoc* (Tukey) test, parents with a Bachelor's degree scored significantly higher in parental responsibility subdimension than those with a high school diploma. However, this effect size was small in magnitude. No significant differences were found across other CFQ subdimensions or FNPA total scores based on parents' education level (all *p* > .05). Similarly, eta-squared values for these comparisons were negligible (*η*^2^ ≤ .01). According to the *post hoc* Tukey test, university graduates had higher scores on the PB factor of the FNPA than parents with a middle-school education. According to the *post hoc* Tukey test, university graduates had higher scores than parents with a middle-school education. These findings suggest that parental education level does not create meaningful or practically significant differences in parental feeding attitudes and behaviours.

**Table 3:** ANOVA results on CFQ and FNPA scores by parents' education level.

Variables	Group	*N*	Mean	*S*	*F*	*P*	Tukey
PR	1. Middle School	85	11.34	2.69	3.13	**.** **04** *	**3–1**
	2. High School	102	10.91	3.02			
	3. Bachelor's Degree	81	11.93	2.44			
	Total	268	11.35	2.77			
PPW	1. Middle School	85	11.57	2.25	.17	.84	—
	2. High School	102	11.72	2.26			
	3. Bachelor's Degree	81	11.54	2.23			
	Total	268	11.62	2.24			
PCW	1. Middle School	85	14.44	3.06	.55	.57	—
	2. High School	102	14.40	3.27			
	3. Bachelor's Degree	81	14.83	2.51			
	Total	268	14.54	2.99			
CCW	1. Middle School	85	8.98	3.20	.03	.96	—
	2. High School	102	8.96	2.54			
	3. Bachelor's Degree	81	8.87	3.03			
	Total	268	8.94	2.90			
RST	1. Middle School	85	26.32	6.71	1.41	.24	—
	2. High School	102	24.69	6.67			
	3. Bachelor's Degree	81	25.62	6.59			
	Total	268	25.49	6.67			
PE	1. Middle School	85	12.56	4.05	.15	.86	—
	2. High School	102	12.50	4.18			
	3. Bachelor's Degree	81	12.23	4.28			
	Total	268	12.44	4.16			
MNT	1. Middle School	85	11.12	3.47	.06	.93	—
	2. High School	102	10.95	3.39			
	3. Bachelor's Degree	81	11.02	3.22			
	Total	268	11.02	3.35			
FNPA Total	1. Middle School	85	58.90	6.94	.41	.65	—
	2. High School	102	59.52	7.36			
	3. Bachelor's Degree	81	59.85	5.82			
	Total	268	59.42	6.78			
PA	1. Middle School	85	15.45	3.22	.03	.96	—
	2. High School	102	15.37	3.15			
	3. Bachelor's Degree	81	15.33	2.97			
	Total	268	15.38	3.11			
PB	1. Middle School	85	11.02	2.00	3.54	**.** **03**	**3–1**
	2. High School	102	11.24	2.02			
	3. Bachelor's Degree	81	11.79	1.63			
	Total	268	11.33	1.92			
UEH	1. Middle School	85	11.25	2.35	.58	.55	—
	2. High School	102	11.27	2.09			
	3. Bachelor's Degree	81	10.95	2.17			
	Total	268	11.17	2.19			
HFI	1. Middle School	85	9.47	1.85	2.46	.08	—
	2. High School	102	9.96	1.52			
	3. Bachelor's Degree	81	9.96	1.68			
	Total	268	9.80	1.69			
SB–RR	1. Middle School	85	11.69	1.90	.12	.87	—
	2. High School	102	11.67	2.16			
	3. Bachelor's Degree	81	11.81	1.67			
	Total	268	11.72	1.93			

* Bold values indicate statistically significant results (*p < 0.05).

Table 4 shows that parents' perceptions and concerns about childhood obesity, as well as their tendencies toward FNPA, do not show a statistically significant difference according to the age of their children (*p* > .05). This suggests that the age of their children does not influence parents' CFQ and FNPA scores. Eta squared (*η*^2^) values ranged between.002 and.024, indicating very small effect sizes. In other words, children's age explained only a negligible proportion of the variance in parents' perceptions, concerns, and feeding practices.

**Table 4:** ANOVA results on parents' CFQ and FNPA scores by children's age groups.

Variables	Group	*n*	Mean	*S*	*F*	*P*
PR	1.8	61	11.49	3.00	.37	.77
	2.9	65	11.24	2.76		
	3.10	67	11.11	2.78		
	4.11	75	11.56	2.61		
	Total	268	11.35	2.77		
PPW	1.8	61	11.88	2.31	.86	.46
	2.9	65	11.29	2.53		
	3.10	67	11.55	2.31		
	4.11	75	11.76	1.82		
	Total	268	11.62	2.24		
PCW	1.8	61	14.32	2.84	.20	.89
	2.9	65	14.73	2.94		
	3.10	67	14.58	2.92		
	4.11	75	14.53	3.24		
	Total	268	14.54	2.99		
CCW	1.8	61	8.95	3.33	.19	.90
	2.9	65	9.16	3.01		
	3.10	67	8.82	2.87		
	4.11	75	8.85	2.48		
	Total	268	8.94	2.90		
RST	1.8	61	24.88	6.09	1.49	.21
	2.9	65	25.40	7.00		
	3.10	67	24.67	7.04		
	4.11	75	26.81	6.41		
	Total	268	25.49	6.67		
PE	1.8	61	12.09	4.46	1.30	.27
	2.9	65	13.26	4.28		
	3.10	67	12.49	4.00		
	4.11	75	11.97	3.90		
	Total	268	12.44	4.16		
MNT	1.8	61	10.68	3.39	.31	.81
	2.9	65	11.06	3.71		
	3.10	67	11.25	3.46		
	4.11	75	11.08	2.92		
	Total	268	11.02	3.35		
FNPA Total	1.8	61	58.62	6.04	1.10	.34
	2.9	65	58.84	6.45		
	3.10	67	60.58	7.62		
	4.11	75	59.56	6.82		
	Total	268	59.42	6.78		
PA	1.8	61	14.63	2.85	1.80	.14
	2.9	65	15.32	3.09		
	3.10	67	15.73	3.48		
	4.11	75	15.74	2.92		
	Total	268	15.38	3.11		
PB	1.8	61	11.86	1.68	2.16	.09
	2.9	65	11.27	1.98		
	3.10	67	11.05	2.14		
	4.11	75	11.21	1.80		
	Total	268	11.33	1.92		
UEH	1.8	61	10.78	2.15	1.51	.21
	2.9	65	11.20	2.39		
	3.10	67	11.59	1.94		
	4.11	75	11.08	2.24		
	Total	268	11.17	2.19		
HFI	1.8	61	9.67	1.71	1.73	.16
	2.9	65	9.49	1.76		
	3.10	67	10.11	1.62		
	4.11	75	9.90	1.63		
	Total	268	9.80	1.69		
SB–RR	1.8	61	11.65	1.89	1.06	.39
	2.9	65	11.55	1.84		
	3.10	67	12.07	2.07		
	4.11	75	11.61	1.91		
	Total	268	11.72	1.93		

Table 5 shows that there was no statistically significant relationship between parents' age and their scores on PR, PPW, PCW, CCW, PE, MNT, FNPA, PA, PB, HFI or SB–RR (all *p* > .05). Age was positively and significantly associated with UEH (*r* = 0.12, *p* < .05) and negatively, albeit weakly, associated with RST (*r* = −.12, *p* < .05), indicating that older parents tend to report slightly healthier eating patterns (fewer unhealthy eating habits) and slightly lower levels of restriction, although both effects are small in magnitude.

**Table 5:** Correlation between parents' age and their CFQ and FNPA scores (*n* = 268).

Variables	PR	PPW	PCW	CCW	RST	PE	MNT	FNPA	PA	PB	UEH	HFI	SB-RR
Age	−.07	−.07	−.00	−.02	−.12*	−.06	−.06	.05	.02	−.06	.12*	.01	.04

Bold values indicate statistically significant correlations (*p < .05).

Table 6 shows the correlations between CFQ subscales and FNPA total and its components. The FNPA total score was positively and significantly correlated with PR and MNT (*r* = .32 and *r* = .41, respectively; *p* < .01), indicating moderate associations: higher FNPA scores are associated with higher PR and MNT scores. However, no significant correlations were observed between FNPA total score and PPW, PCW, CCW, RST or PE (all *p* > .05).

**Table 6:** Correlation between CFQ subscales and FNPA total score (*n* = 268).

Variables	PR	PPW	PCW	CCW	RST	PE	MNT
FNPA Total	**.** **32** **	.01	.06	.00	.00	.09	**.** **41** **
PA	**.** **24** **	.03	.09	.01	.07	.10	**.** **29** **
PB	**.** **28** **	.00	.00	.10	.07	.08	**.** **26** **
UEH	.03	−.05	−.06	−.10	**−**.24**	−.11	.03
HFI	**.** **19** **	.07	.11	−.02	.04	.11	**.** **24** **
SB-RR	.25**	−.01	.06	.01	.04	.11	**.** **45** **

Bold values indicate statistically significant correlations; **p < .01.

When FNPA components were examined, PA showed positive, significant correlations with PR *(r* = .24, *p* < .01) and MNT (*r* = .29, *p* < .01), suggesting that higher family physical activity levels are associated with greater parental involvement and monitoring of the child's eating. Similarly, PB was positively and significantly correlated with PR (*r* = .28, *p* < .01) and MNT (*r* = .26*, p* < .01), indicating that in families with more favourable eating patterns, PR and MNT tend to be higher. UEH was negatively and significantly associated with RST (*r* = −.24, *p* < .01), implying that families who report fewer unhealthy eating habits also tend to use less restrictive feeding practices; correlations between UEH and the other CFQ subscales were not statistically significant. HFI showed positive, significant correlations with PR (*r* = .19, *p* < .01) and MNT (*r* = .24, *p* < .01), indicating that healthier eating behaviours are associated with higher PR and MNT. SB–RR was positively and significantly correlated with PR (*r* = .25, *p* < .01) and especially with MNT (*r* = .45, *p* < .01), the latter reflecting a moderate association and suggesting that parental monitoring of children's sedentary behaviours is strongly related to family practices captured by FNPA. Overall, these findings show that FNPA total and FNPA components exhibit consistent and meaningful associations primarily with PR and MNT, whereas relationships with PPW, PCW, CCW, PE and RST are more limited, suggesting that family-based healthy lifestyle behaviours are more closely linked to active parental involvement and monitoring than to parental weight perceptions or more controlling feeding strategies.

Table 7 shows that the regression model predicting FNPA scores was significant (*F* = 12.64, *p* < .001). The model explained approximately one quarter of the variance in FNPA scores (*R*^2^ = .25; Adj. *R*^2^ = .23), and the Durbin–Watson coefficient (1.88) indicated no serious autocorrelation. PR (*β* = .22, *p* < .001) and MNT (*β* = .43, *p* < .001) were positively and significantly associated with FNPA scores, whereas RST (*β* = −.17, *p* < .01) showed a negative association. PPW, PCW, CCW and PE did not significantly contribute to the model (all *p* > .05). These findings suggest that higher levels of PR and MNT tend to co-occur with a more favourable family nutrition and physical activity environment, while greater use of RST tends to be linked to a less favourable environment, although the cross-sectional design does not permit causal inferences.

**Table 7:** Prediction of FNPA scores by CFQ subdimensions.

Variables	*B*	SE_B_	*β*	*t*	*p*	Tolerance	VIF
Constant	48.09	2.93		16.39	**.** 00*		
PR	.55	.14	.22	3.88	**.** 00*	.85	1.17
PPW	.02	.17	.00	.12	.89	.88	1.13
PCW	.09	.12	.04	.75	.44	.90	1.10
CCW	−.17	.13	−.07	−1.26	.20	.80	1.24
RST	−.17	.06	−.17	−2.64	**.** 00*	.65	1.51
PE	−.01	.09	−.00	−.12	.89	.78	1.26
MNT	.87	.12	.43	6.95	**.** 00*	.73	1.35
*R* = .504	*R*^2^ = .254 Adj. *R*^2^ = .234			DW = 1.88		*F* = 12.637, *p* = .00*	

Bold *p* values indicate statistically significant predictors (*p < .05).

## Discussion

This study examined how parental perceptions and feeding practices are associated with families' tendencies toward healthy nutrition and physical activity, using validated measures of feeding style and the home environment. Overall, the findings contribute to a more nuanced understanding of how responsibility, monitoring, and control-based practices relate to family lifestyle patterns during middle childhood.

A central result was the positive and statistically significant association between the CFQ subdimensions of parental PR and MNT and the FNPA total score. Parents who reported assuming greater responsibility for their child's eating and who more consistently monitored eating behaviours tended to report home environments characterised by more health-promoting nutrition and physical activity routines. This pattern is consistent with previous research showing that parental engagement in everyday practices—such as preparing balanced meals, organising opportunities for movement and managing screen time—is closely linked to healthier child behaviours (25, 45–47). Parental monitoring can be conceptualised as a form of behavioural regulation that includes supervision, limit-setting and modelling of desired behaviours, and when applied in a structured but non-coercive manner, it is typically associated with more favourable outcomes and reduced exposure to obesogenic influences (24, 48). In the present regression model, PR and MNT remained positively associated with FNPA scores, whereas RST showed a negative association, with the model explaining approximately one quarter of the variance in FNPA. This represents a moderate effect size and indicates that feeding perceptions and practices are important, though not exclusive, correlates of the family health environment.

In line with this pattern at the total-scale level, the examination of FNPA components showed that PA, PB, HFI and SB–RR were all positively related to PR and MNT. These findings suggest that families who report more active lifestyles, more favourable eating patterns and more structured management of sedentary time also tend to report higher levels of parental responsibility and monitoring in feeding. By contrast, UEH were negatively associated with RST, indicating that families with fewer unhealthy dietary practices report lower levels of restrictive feeding, and vice versa. Overall, FNPA total and domain scores displayed consistent and meaningful associations primarily with PR and MNT, whereas relationships with parental weight perceptions (PPW, PCW, CCW), PE and RST were more limited.

Interestingly, no significant bivariate correlations were observed between FNPA total scores and the CFQ subscales reflecting restriction and pressure to eat, although RST emerged as a negative predictor in the multivariable regression. RST and PE capture more controlling feeding strategies that have often been linked to less adaptive eating patterns in children. The absence of positive associations with FNPA suggests that families with generally healthier lifestyle patterns do not necessarily rely on restrictive or coercive approaches. Previous studies have indicated that strong emphasis on restriction or pressure to eat can undermine children's internal hunger and satiety cues, increase resistance and contribute to dysregulated eating (49–51). The current findings are compatible with a gradual shift towards more autonomy-supportive approaches in at least part of the sample, or with an increasing recognition that highly controlling feeding practices are not well aligned with long-term health promotion. At the same time, the negative association between RST and FNPA in the regression analysis indicates that, when considered alongside other feeding dimensions, higher use of restriction tends to co-occur with less favourable family nutrition and activity patterns.

No meaningful gender differences were identified for parents or children in terms of CFQ or FNPA scores, and effect size estimates indicated that the influences of gender, education and child age on parental perceptions and feeding practices were negligible in practical terms. This contrasts with earlier studies reporting that mothers often assume more responsibility and express greater concern about child feeding than fathers (52, 53). One possible explanation is that parental roles in contemporary families are becoming more egalitarian, with mothers and fathers more frequently sharing caregiving and health-related responsibilities. It is also plausible that public health campaigns and widely available digital resources have encouraged both parents, regardless of gender, to adopt similar roles in supporting healthy lifestyles.

With respect to parental education, a statistically significant difference emerged only for the PR subdimension, with university graduates reporting higher responsibility scores than high school graduates. This finding is broadly consistent with evidence linking higher educational attainment to greater engagement in health-promoting behaviours (54, 55). However, the absence of systematic differences in other CFQ subdimensions and FNPA scores across educational groups may indicate that basic nutrition and activity messages are increasingly reaching a wide range of parents, potentially diminishing educational gradients in some aspects of family practice. Public health initiatives, school-based programmes and online health information may collectively contribute to a more generalised awareness of healthy lifestyles that is less tightly confined to formally educated groups.

Child age was not associated with significant variation in CFQ or FNPA scores, suggesting that parents in this sample maintained broadly similar perceptions and feeding practices across the middle-childhood age range (56, 57). This period is often characterised by relatively stable family routines, where parents continue to play a central role in organising daily schedules, and peer and school influences, while emerging, may not yet fully reshape health behaviours (58, 59). A small but noteworthy pattern was the negative correlation between parental age and RST, indicating that older parents tended to report slightly lower levels of restrictive feeding. This may reflect increased confidence and experience in managing children's eating, a preference for more flexible or communication-based strategies, or a shift towards guidance rather than strict control as parents age.

These patterns should also be interpreted within the cultural context of Türkiye. Turkish families are often characterised by close intergenerational ties and a strong parental role in structuring children's daily lives, including meal routines and opportunities for physical activity. Such norms may support high levels of responsibility and monitoring, but can also co-exist with more controlling strategies, such as restriction and pressure to eat. The combination of relatively high monitoring and restriction observed in this study resembles patterns reported in previous Turkish research on parental feeding, and differs to some extent from findings in Western samples where monitoring is more clearly differentiated from overt control. Cultural expectations related to hospitality, generous portion sizes and respect for parental authority may help to explain why supportive and controlling practices can be present within the same families and may account for some discrepancies with studies conducted in other sociocultural settings.

Taken together, the findings suggest that parental involvement and supervision—particularly through responsibility and monitoring—are more consistently associated with favourable family nutrition and physical activity patterns than control-based strategies such as restriction or pressure to eat. For practitioners and intervention designers, this underscores the importance of encouraging parents to be engaged, informed and responsive rather than overly coercive. Family-centred programmes that strengthen parents' sense of responsibility, enhance monitoring skills and support the creation of structured yet autonomy-supportive environments may be especially valuable. Given the limited influence of gender and most demographic characteristics in this sample, such interventions may be applicable across a broad range of family structures in similar urban contexts.

Several limitations should be considered when interpreting these results. The purposive sampling strategy restricts generalisability, as parents who chose to participate may not be representative of the wider parent population. Data were collected solely through self-report instruments, which are vulnerable to recall bias and socially desirable responding. Detailed socioeconomic indicators (e.g., household income, occupational status, neighbourhood characteristics) were not obtained, despite socioeconomic position being a well-established determinant of dietary patterns, physical activity and family health behaviours. The absence of these variables limits the ability to examine potential confounding or moderating effects of socioeconomic circumstances. In addition, no objective measures of children's weight status or physical activity (such as BMI or accelerometer-based indices) were included, which constrains validation of parental reports and prevents direct linkage of family patterns to anthropometric or behavioural outcomes. Although validated instruments (CFQ and FNPA) were used and internal consistency values in this sample were acceptable, other psychometric properties were not comprehensively re-evaluated. Finally, the cross-sectional design precludes causal inferences. Longitudinal and multi-site studies that incorporate detailed socioeconomic data, objective indicators of child weight and activity, and richer assessments of family structure and context would allow more precise examination of the pathways linking parental perceptions and feeding practices to family health behaviours and child outcomes, and would support the development of culturally sensitive, family-based strategies to promote healthier lifestyle patterns in middle childhood.

## Conclusion

This study examined how parental perceptions and feeding practices are associated with families' tendencies toward healthy nutrition and physical activity, showing that higher levels of parental responsibility and monitoring co-occur with more favourable family patterns, whereas more controlling strategies, such as restriction, tend to display weaker or inverse associations. Differences by parent and child gender, age and education level were small, suggesting that supportive and controlling feeding practices are relatively widespread across parent groups in this urban context in Türkiye rather than confined to specific sociodemographic strata. Overall, the findings underline the importance of parenting behaviours that combine structured involvement and active supervision with a non-coercive approach to children's eating, and they point to the value of interventions that strengthen parental responsibility and engagement in everyday routines while discouraging overly restrictive or pressuring feeding practices. Future work using longitudinal and multi-site designs, including detailed socioeconomic indicators, objective measures of children's weight status and physical activity, and richer assessments of family structure and context, would help to clarify the pathways linking parental perceptions and feeding practices with family health behaviours and support the development of culturally sensitive, family-based strategies to promote healthier lifestyle patterns in middle childhood.

## Data Availability

The raw data supporting the conclusions of this article will be made available by the authors, without undue reservation.
